# Thy-1 Attenuates TNF-α-Activated Gene Expression in Mouse Embryonic Fibroblasts via Src Family Kinase

**DOI:** 10.1371/journal.pone.0011662

**Published:** 2010-07-19

**Authors:** Bin Shan, James S. Hagood, Ying Zhuo, Hong T. Nguyen, Mark MacEwen, Gilbert F. Morris, Joseph A. Lasky

**Affiliations:** 1 Department of Medicine, Tulane University Health Sciences Center, New Orleans, Louisiana, United States of America; 2 Department of Pediatrics, University of Alabama-Birmingham School of Medicine, Birmingham, Alabama, United States of America; 3 Department of Pathology, Tulane University Health Sciences Center, New Orleans, Louisiana, United States of America; University of Chicago, United States of America

## Abstract

Heterogeneous surface expression of Thy-1 in fibroblasts modulates inflammation and may thereby modulate injury and repair. As a paradigm, patients with idiopathic pulmonary fibrosis, a disease with pathologic features of chronic inflammation, demonstrate an absence of Thy-1 immunoreactivity within areas of fibrotic activity (fibroblast foci) in contrast to the predominant Thy-1 expressing fibroblasts in the normal lung. Likewise, Thy-1 deficient mice display more severe lung fibrosis in response to an inflammatory injury than wildtype littermates. We investigated the role of Thy-1 in the response of fibroblasts to the pro-inflammatory cytokine TNF-α. Our study demonstrates distinct profiles of TNF-α-activated gene expression in Thy-1 positive (Thy-1+) and negative (Thy-1−) subsets of mouse embryonic fibroblasts (MEF). TNF-α induced a robust activation of MMP-9, ICAM-1, and the IL-8 promoter driven reporter in Thy-1− MEFs, in contrast to only a modest increase in Thy-1+ counterparts. Consistently, ectopic expression of Thy-1 in Thy-1− MEFs significantly attenuated TNF-α-activated gene expression. Mechanistically, TNF-α activated Src family kinase (SFK) only in Thy-1− MEFs. Blockade of SFK activation abrogated TNF-α-activated gene expression in Thy-1− MEFs, whereas restoration of SFK activation rescued the TNF-α response in Thy-1+ MEFs. Our findings suggest that Thy-1 down-regulates TNF-α-activated gene expression via interfering with SFK- and NF-κB-mediated transactivation. The current study provides a novel mechanistic insight to the distinct roles of fibroblast Thy-1 subsets in inflammation.

## Introduction

Thy-1 is a glycosyl phosphatidylinositol-anchored glycoprotein that is expressed in a variety of cell types, including T cells, thymocytes, neurons, endothelial cells, and fibroblasts [1], [2], [3]. Thy-1 is involved in T cell activation, inflammation, wound healing, and fibrosis. To mediate these diverse effects, Thy-1 participates in multiple signaling cascades. One of numerous Thy-1 interacting signaling molecule is Src Family Kinase (SFK). After T cell receptor activation, thymocytes isolated from Thy-1 null mice have increased SFK activity and cell proliferation when compared with thymocytes collected from wild-type mice, which indicates that Thy-1 inhibits T cell receptor-induced SFK activation and proliferation of thymocytes [4]. On the other hand, SFK is transiently activated by thrombospondin-1 in Thy-1+, but not in Thy-1− rat lung fibroblasts [5]. These contradictory reports implicate that Thy-1 modulates SFK activity in a manner dependent upon cellular context.

Accumulating evidence indicates that fibroblasts are important modulators of inflammation and immunity [6], [7], [8]. One intriguing phenomenon observed in fibroblasts is their phenotypic heterogeneity based on surface expression of Thy-1 and its subsequent pathological implications associated with inflammation [9], [10], [11], [12]. Thy-1 appears to regulate fibroblast responses to inflammatory cytokines. Thy-1 subpopulations of orbital fibroblasts differ in PGE2 and IL-8 production, in that IL-1β induces greater PGE2 expression in Thy-1+ fibroblasts, but stimulates more IL-8 in Thy-1− fibroblasts [13], [14]. Thy-1 subpopulations of primary rat lung fibroblasts exhibit distinct proliferative responses, signal transduction, and cell morphology upon exposure to PDGF-AA and IL-1β [15], [16], [17]. Thy-1− rat lung fibroblasts also possess a greater ability to activate latent TGF-β1 in response to pro-fibrotic cytokines and bleomycin than do their Thy-1+ counterparts [18].

Immunohistochemical analyses of human lung sections reveal that fibroblasts within fibroblastic foci of idiopathic pulmonary fibrosis do not express Thy-1, whereas most fibroblasts from normal lungs are Thy-1+ [19]. In a murine lung model of bleomycin-induced fibrosis, Thy-1 deficient mice develop more severe lung fibrosis in comparison to wildtype littermates [19]. These findings underscore the biological significance of fibroblast-expressed Thy-1 in inflammation-associated fibrogenesis. However, the molecular mechanism underlying Thy-1 modulated inflammatory responses remains largely unknown.

TNF-α is a major pro-inflammatory cytokine. Upon binding to its receptors on the cell membrane, TNF-α initiates an inflammatory cascade consisting of increases in COX-2, iNOS, and endothelial adhesion molecule expression, recruitment of inflammatory cells and eventual tissue destruction [20]. TNF-α activates expression of a panel of inflammation modulators and executors via activating transcription factors such as NF-κB and AP-1. Resident fibroblasts in various organs are a major target cell type for TNF-α to modulate inflammation. For instance, TNF-α primes lung fibroblasts to produce Th1 type chemokines in interstitial lung diseases [21], and in rheumatoid arthritis, TNF-α stimulates secretion of matrix metalloproteinases by synovial fibroblasts [22]. Among the signaling pathways activated by TNF-α, SFK activation is required for activation of NF-κB and the consequent transcriptional activation of TNF-α target genes, such as ICAM-1 and COX-2 in human epithelial cell lines [23], [24].

The current study investigates whether Thy-1 expression regulates gene activation by TNF-α in fibroblasts. Our results indicate that Thy-1 expression in mouse embryonic fibroblasts (MEF) substantially attenuates gene activation by TNF-α, which includes MMP-9, ICAM-1, and a reporter gene controlled by the human IL-8 promoter. Moreover, SFK activation is required for TNF-α-activated gene expression in MEFs. Our findings further suggest that Thy-1 interferes with SFK activation by TNF-α and thereby inhibits TNF-α-activated gene expression in MEFs.

## Results

### Differential expression of the TNF-α-activated genes in MEF Thy-1 subsets

**Figure 1:**
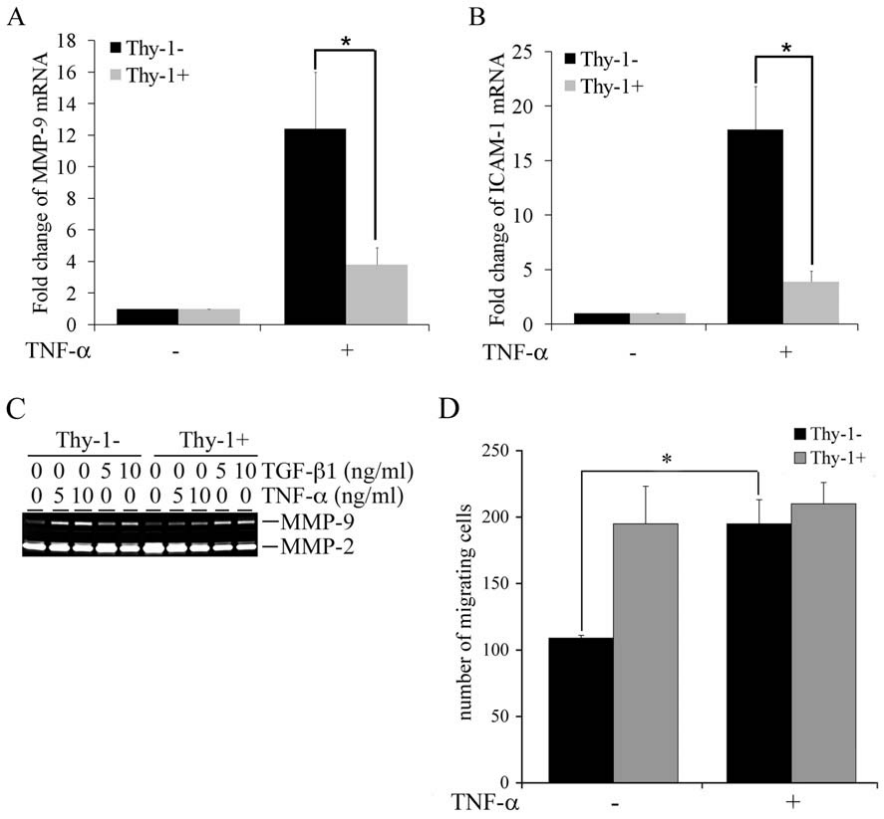
Differential Expression of MMP-9 and ICAM-1 in TNF-α-stimulated MEF Thy-1 subsets. A) Serum starved Thy-1+ and Thy-1− MEFs were stimulated with TNF-α (5 ng/ml) for 24 hours. Total RNA was isolated from the stimulated MEFs. Quantitative RT-PCR was carried out to determine the mRNA levels of MMP-9. The fold change of MMP-9 mRNA in the stimulated cells over control was obtained by setting the values from the untreated MEFs to one. B) The culture conditions were similar to part A except that the mRNA levels of ICAM-1 were determined. C) The conditioned medium was collected from MEFs exposed to the indicated doses of TNF-α and TGF-β1 for 48 hrs. Gelatin zymography was carried out to determine the expression MMP-2/-9 in the conditioned medium. A representative gel image from three experiments was shown. D) Thy-1 MEF subsets were seeded into Matrigel-coated transwell chambers. The cells were stimulated by TNF-α (5 ng/ml) for 24 hours. The invading cells on the bottom surface of the membrane were stained and enumerated under light microscopy at 400× amplification. The sum of invading cells from 5 randomly selected fields was compared across the groups. The invasion assays were performed in triplicates for each treatment group. Results shown were mean and standard deviations from at least three independent experiments. *, P value<0.05.

**Figure 2:**
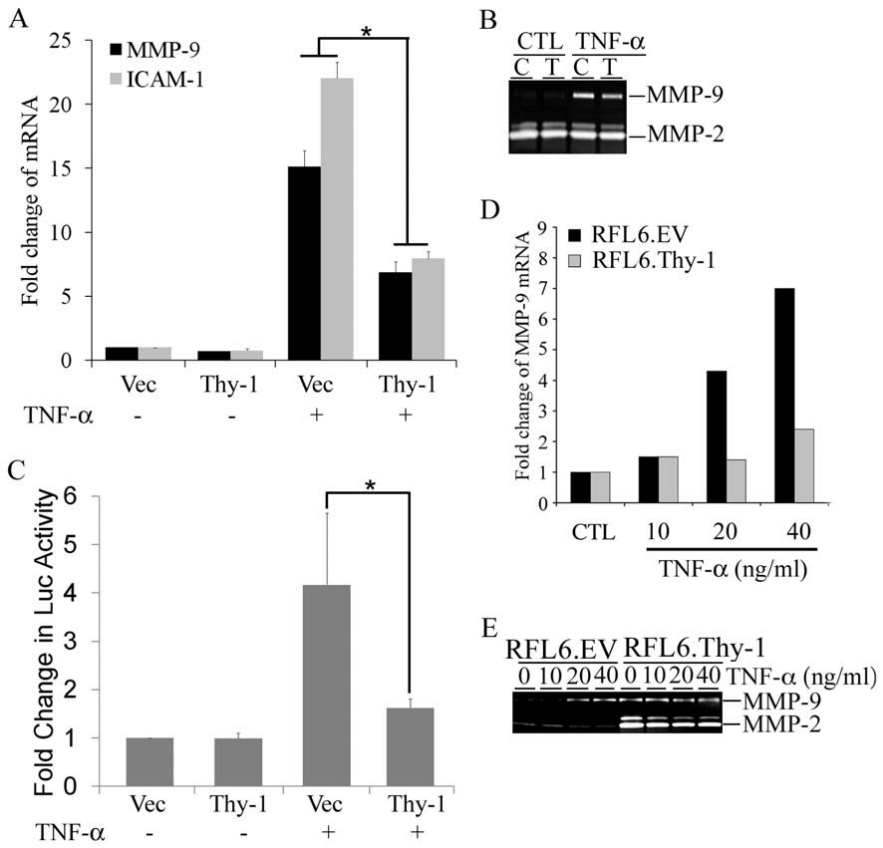
Reduced TNF-α-activated gene expression by Thy-1 expression in Thy-1– fibroblasts. A) Thy-1– MEFs were transiently transfected with either the Thy-1 expression vector (Thy-1) or the backbone vector (Vec) followed by exposure to TNF-α (5 ng/ml) for 24 hrs. Total RNA was isolated from the stimulated MEFs. Quantitative RT-PCR was carried out to determine the mRNA levels of MMP-9 and ICAM-1. The fold change of each mRNA in the stimulated cells over control was obtained by setting the values from the untreated backbone vector transfected Thy-1– MEFs to one. B) The culture conditions were similar to part A except that the transfected Thy-1– MEFs were exposed to TNF-α (5 ng/ml) for 48 hrs. The conditioned medium was collected and analyzed for the expression of MMP-2/-9 by gelatin zymography. A representative gel image from three experiments was shown. C) The culture conditions were similar to part A except that the IL-8-Luc and RL-TK reporters were co-transfected with Thy-1 or the backbone vector. A fold increase in IL-8-Luc by TNF-α (5 ng/ml) was determined by setting the values from the unstimulated Thy-1– MEFs to one. D) The serum-starved RLE6 variants were stimulated with the indicated doses of TNF-α for 24 hrs. The mRNA levels of MMP-9 were determined as described in part A. The results were average from two independent experiments. E) Similar to Part D except that MMP-9 expression in the conditioned medium was determined using gelatin zymogram in RLE6 variants stimulated with TNF-α for 48 hrs. The gel image is representative of two independent experiments. Vec and V refer to Thy-1– MEFs transfected with the backbone vector. Thy-1 and T refer to Thy-1– MEFs transfected with the Thy-1 expression vector. In parts A & C, the results shown are the mean and standard deviation from at least three independent transfections. *, P value<0.05.

Thy-1 expression has been implicated in regulating inflammatory responses in fibroblasts. Since TNF-α is a major pro-inflammatory cytokine, we questioned whether Thy-1 regulates gene activation by TNF-α in fibroblasts. Sorted subsets of MEFs based on surface expression of Thy-1 (Thy-1− and Thy-1+) were serum-starved and exposed to TNF-α (5 ng/ml) for 24 hours. Expression of MMP-9 and ICAM-1, two TNF-α-activated genes, were determined by quantitative RT-PCR. TNF-α induced a robust induction of MMP-9, a 12-fold increase over the control group in Thy-1− MEF, in contrast to a modest 4-fold increase in Thy-1+ MEF (Figure 1A). The differential MMP-9 response to TNF-α emerged as early as 12 hours post exposure with greater disparity between Thy-1 subsets, a 33-fold increase over control for Thy-1− MEF *vs.* a 6-fold increase over control for Thy-1+ MEF (data not shown). A similar profile was observed in ICAM-1 with a 18-fold increase in the mRNA levels of ICAM-1 in TNF-α stimulated Thy-1− MEF *vs.* a 4-fold increase in Thy-1+ MEF (Figure 1B). To confirm the difference in MMP-9 induction in response to TNF-α, expression of MMP-9 was examined using gelatin zymography in the conditioned medium collected from the TNF-α stimulated MEFs. Consistently, MMP-9 expression in the conditioned medium exhibited distinct TNF-α (5 and 10 ng/ml) responses t between Thy-1 subsets. MMP-9 expression was substantially induced by TNF-α in Thy-1− MEFs with a more than 3-fold increase over control as determined by densitometry, whereas in comparison there was only a minimal change in MMP-9 in Thy-1+ MEF (Figure 1C). In contrast, MMP-9 protein was induced to a similar extent by TGF-β1 (5 and 10 ng/ml) in both subsets (Figure 1C). The TGF-β1-induced increase in MMP-9 mRNA levels was also comparable between the Thy-1 subsets, a 10-fold increase in Thy-1− MEFs vs. a 12-fold increase in Thy-1+ MEFs in response to 5 ng/ml of TGF-β1 (data not shown). Neither TNF-α nor TGF-β1 altered MMP-2 expression in the conditioned medium of MEF Thy-1 subsets (Figure 1C). More importantly, only Thy-1− MEFs responded to TNF-α with an increase in invasion through a Matrigel barrier, although Thy-1+ MEFs exhibited greater baseline invasiveness (Figure 1D). The higher baseline of invasion in Thy-1+ MEFs correlated with a higher basal expression of MMP-2 at both mRNA and protein levels (Figure 1, C & D, and data not shown). To validate the Thy-1-mediated regulation of TNF-α-activated gene expression, we transiently transfected Thy-1− MEFs with either a backbone vector or a Thy-1 expressing vector. The transient transfection resulted in a 30-fold increase in Thy-1 mRNA in Thy-1− MEFs (data not shown). In agreement with the results from MEF Thy-1 subsets, expression of Thy-1 in Thy-1− MEFs substantially reduced TNF-α-induced MMP-9 and ICAM-1 to less than 50% of the increase observed in Thy-1− MEF transfected with the backbone vector (Fig. 2A). Moreover, TNF-α-induced MMP-9 expression in the conditioned medium was also significantly reduced (Fig. 2B). Since TNF-α signaling can transcriptionally activate its target genes, including MMP-9, ICAM-1, and IL-8 through activation of transcription factor NF-κB and AP-1, we questioned whether Thy-1 expression regulates transcriptional activation of the TNF-α target promoters [25]. We used the human IL-8 promoter reporter which harbors the NF-κB and AP-1 response elements [26]. Indeed, TNF-α induced a 4-fold increase of the reporter activity in Thy-1− MEFs transfected with the backbone vector, whereas TNF-α-mediated activation of the IL-8 promoter was diminished by Thy-1 expression as demonstrated by only a marginal increase of only 1.5-fold in Thy-1− MEFs transfected with Thy-1 (Fig. 2C). Thy-1-modulated TNF-α-activated gene expression was also assessed in variants of a rat fetal lung fibroblast line, the Thy-1− (RLE6.EV) and the Thy-1 overexpressing (RLE6.Thy-1). MMP-9 induction by TNF-α was examined by qRT-PCR (Figure 2D) and gelatin zymography (Figure 2E). Similar to Thy-1− MEFs, RLE6.EV responded to TNF-α with a remarkable increase in MMP-9 (Figure 2, D & E). MMP-9 expression remained unchanged upon exposure TNF-α in the RFL6.Thy-1 cells. However, RFL6.Thy-1 cells exhibited much greater basal MMP-2 and MMP-9 expression than that of RFL6.EV cells (Figure 2E and data not shown). Nevertheless, with respect to the TNF-α-activated gene expression, the results from RFL6.Thy-1 variants corroborated the observations from MEF Thy-1 subsets in that Thy-1 expression attenuated TNF-α-activated gene expression in fibroblasts.

### SFK activation in TNF-α-stimulated MEF Thy-1 subsets

**Figure 3:**
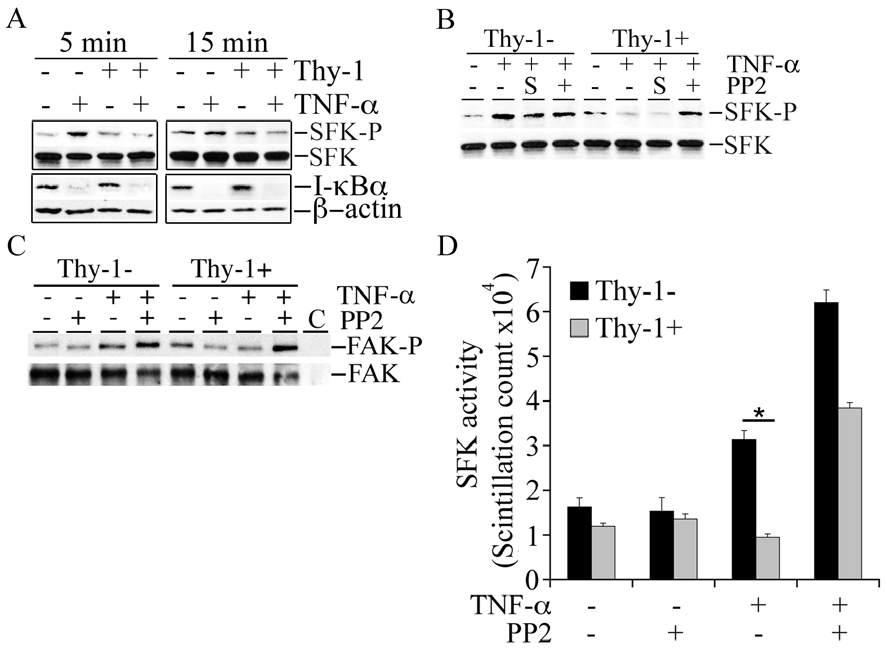
TNF-α-mediated activation of SFK in MEF Thy-1 subsets. A) MEF Thy-1 subsets were exposed to TNF-α (5 ng/ml) for 5 and 15minutes. The treated MEFs were lysed and immunoblotted for tyrosine 416 phosphorylated-SFK, total SFK, I-κBα, and β-actin. B) Similar to part A except that MEF Thy-1 subsets were pretreated with PP2 (10 µM) for 30 minutes, and then exposed to TNF-α (5 ng/ml) for 5 minutes. The cell lysates were immunoblotted for tyrosine 416 phosphorylated-SFK and total SFK. C) Serum-starved MEFs were exposed to TNF-α (5 ng/ml) ± PP2 (10 µM) for 5 minutes and cell lysates were immunoprecipitated with an SFK-specific antibody. The level of phosphorylated FAK in SFK-specific antibody immunoprecipitates was measured by immunoblots with a phosphotyrosine-specific antibody. The blot was then stripped and reprobed with a FAK-specific antibody to determine the amount of SFK-associated FAK. D) The MEFs were treated and immunoprecipitated with an SFK-specific antibody as in part C. SFK activity was determined using in vitro SFK assay as described in Methods. Results shown were mean and standard deviations from three independent experiments. *, P value<0.05. The results represent the average and standard error from three separate assays. The immunoblots in parts A to C are representative of two independent experiments. “SFK-P” refers to SFK with tyrosine 416 phosphorylated; “FAK-P” refers to FAK with tyrosine residues phosphorylated.

**Figure 4 pone-0011662-g004:**
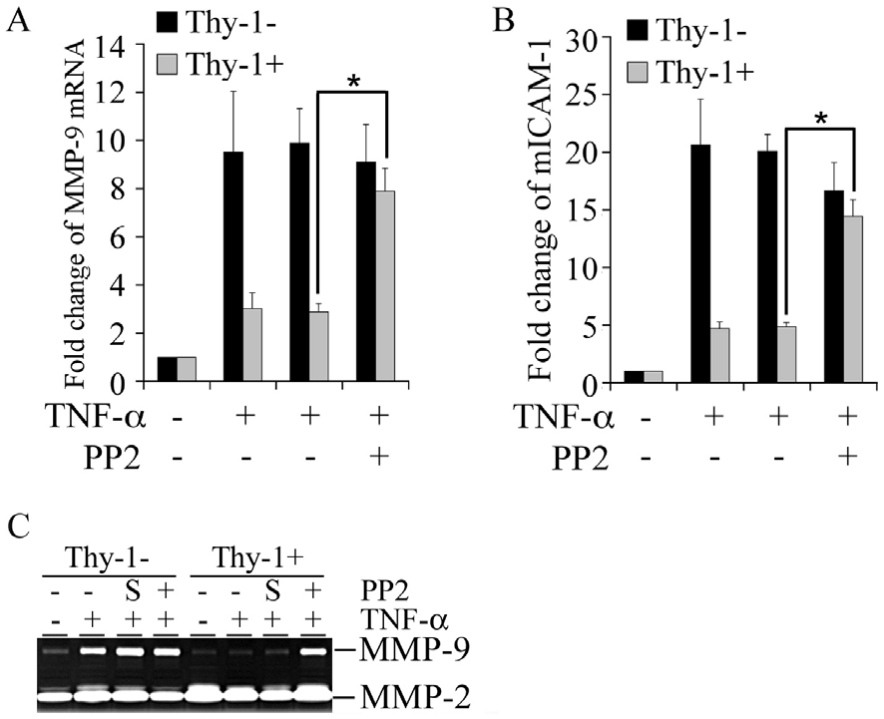
Rescue of TNF-α-activated gene expression by PP2 in Thy-1+ MEFs. A) Serum-starved MEFs were pre-treated with either DMSO (solvent control) or PP2 (10 µM) for 30 minutes followed by exposure to TNF-α (5 ng/ml) for 24 hours. Total cellular RNA was isolated and quantitative RT-PCR was performed to determine the mRNA levels of MMP-9. The fold change of each mRNA was obtained by correction with the corresponding values of the house keeping gene 36B4 and by setting the values from the control group to one. B) Similar to part A except that the mRNA levels of ICAM-1 was determined by quantitative RT-PCR. C) MEF Thy-1 subsets were treated as in part A for 48 hrs. The conditioned medium was collected from each treatment group and processed for gelatin zymography to determine the expression of MMP-9 in the conditioned medium. “S” refers to the solvent DMSO. The results represent the mean and standard deviation from three independent experiments. *, P value<0.05.

Several studies have documented that Thy-1 regulates SFK activity [5], [27], [28], [29]. Activation of SFK plays a critical role in TNF-α signaling in epithelial cells [23], [24]. To explore the potential interaction between Thy-1 and SFK activation by TNF-α in MEFs, SFK activity was examined by immunoblots with an antibody specific for the active form of SFK which is marked by phosphorylation of tyrosine 416 in c-Src as well as the equivalent sites in other SFKs. TNF-α stimulated a transient increase in phosphorylation of SFK only in the Thy-1− subpopulation, which emerged at 5 minutes post exposure and declined to baseline by 15 minutes (Figure 3A, top panel). On the contrary, TNF-α did not induce a noticeable change in SFK phosphorylation in Thy-1+ MEFs (Figure 3A, top panel). Immunoblots for SFK protein from the same blots did not reveal any significant difference in protein levels of SFK between two subpopulations (Figure 3A, top panel). Since degradation of I-κBα and the consequent activation of NF-κB are pivotal steps for TNF-α-activated gene expression [23], [24], we examined whether Thy-1 modulates degradation of I-κBα. The baseline of the protein levels of I-κBα was similar in the MEF Thy-1 subpopulations. In addition, the TNF-α-induced degradation in I-κBα was indistinguishable between the Thy-1 subsets through 15 minutes post exposure (Figure 3A, bottom panel). We next sought to determine the role of SFK activation in the distinct response of MEF Thy-1 subsets to TNF-α by employing PP2, a SFK selective small molecule inhibitor. MEFs were pretreated with PP2 (10 µM) for 30 minutes followed by exposure to TNF-α (5 ng/ml). Surprisingly, PP2 (10 µM) rescued SFK activation in TNF-α stimulated Thy-1+ MEFs, whereas PP2 exhibited little effect on SFK phosphorylation in Thy-1− MEFs (Figure 3B). Moreover, TNF-α stimulated phosphorylation of FAK associated with SFK only in Thy-1− MEFs (Figure 3C). Because FAK is a well documented substrate of SFK, elevated phosphorylation of SFK-associated FAK further supported activation of SFK in Thy-1− MEFs. More importantly, PP2 increased phosphorylation of SFK-associated FAK in Thy-1+ MEFs exposed to TNF-α (Figure 3C), which was consistent with SFK activation by PP2 in Thy-1+ MEFs (Figure 3B). SFK activity in the MEFs exposed to the above treatments was further directly measured *in vitro* using a Src Assay Kit as described in Methods. TNF-α stimulated SFK-associated phosphorylation of SFK-specific peptide substrate only in Thy-1− MEF, whereas a combination TNF-α and PP2 activated SFK in both Thy-1 subsets (Figure 3D). The *in vitro* kinase assays confirmed that PP2, at least in MEFs under the current experimental conditions, activated rather than inhibited SFK activity. Consequently, PP2 rescued MMP-9 and ICAM-1 induction by TNF-α in Thy-1+ MEF to a level comparable to that in Thy-1− MEF (Figure 4, A and B). Rescued induction of MMP-9 was further confirmed by MMP-9 expression in the conditioned medium collected from Thy-1+ MEFs exposed to TNF-α (Figure 4C).

### Requirement of SFK activation for TNF-α-activated gene expression in MEFs

**Figure 5 pone-0011662-g005:**
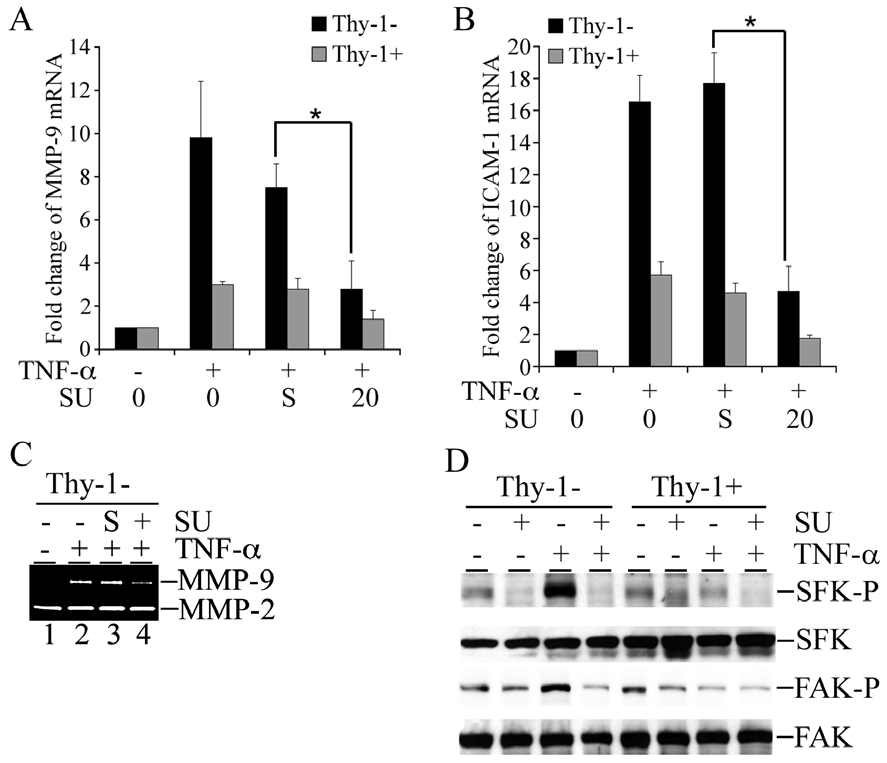
Abrogation of TNF-α-activated gene expression by SFK blockade. A & B) Serum-starved MEFs were pre-treated with either DMSO (solvent control) or SU6656 (20 µM) for 30 minutes followed by exposure to TNF-α (5 ng/ml) for 24 hours. Total cellular RNA was isolated and subject to quantitative RT-PCR for MMP-9 and ICAM-1 as described in Methods. The fold change of each mRNA was obtained by correction for the corresponding values of the house keeping gene 36B4 and by setting the values from the control group to one. The results were presented in means and standard deviations from three independent experiments. *, P value<0.05. C) The conditioned medium was collected from each treatment group of MEFs as in parts A & B and processed for gelatin zymography as described in Methods. D) Serum-starved MEF were pretreated with SU6656 (20 µM) for 30 minutes followed by exposure to TNF-α for 5 minutes. The exposed cells were then lysed and immunoblotted for tyrosine 416 phosphorylated-SFK, total SFK. The level of phosphorylated FAK in SFK-specific antibody immunoprecipitates was measured by immunoblots using a phosphotyrosine-specific antibody. The blot was then stripped and reprobed with a FAK-specific antibody to determine the amount of SFK-associated FAK. “SFK-P” refers to SFK with tyrosine 416 phosphorylated; “FAK-P” refers to FAK with tyrosine residues phosphorylated. A representative image from at least two independent experiments is presented in parts C and D.

**Figure 6 pone-0011662-g006:**
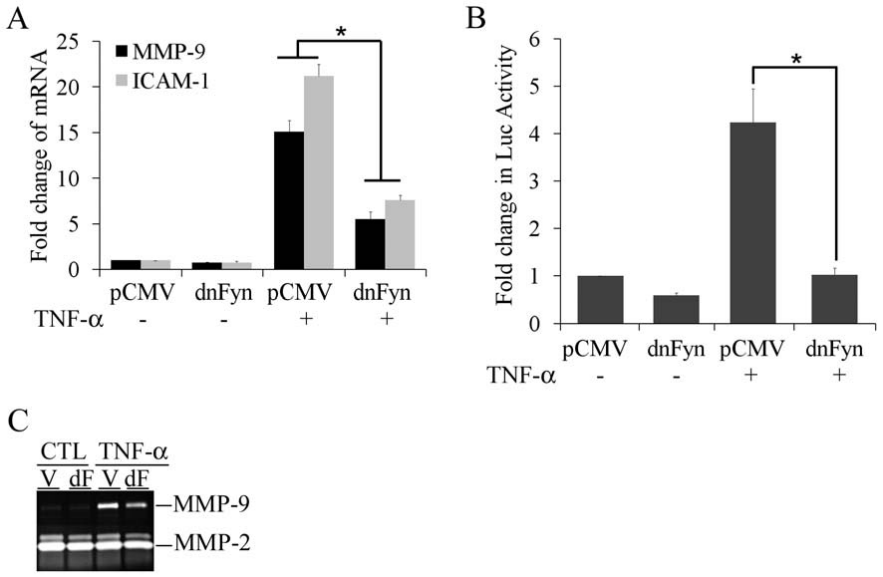
Attenuated TNF-α-activated gene expression by dnFyn in Thy-1– MEF. The dnFyn expression vector or the backbone vector was transiently transfected into Thy-1– MEF prior exposure to TNF-α. A & B) Serum-starved MEFs were exposed to TNF-α (5 ng/ml) for 24 hours. Total cellular RNA was isolated and subject to quantitative RT-PCR for MMP-9 and ICAM-1, respectively. The fold change of each mRNA was obtained by correction for the corresponding values of the house keeping gene 36B4 and by setting the values from the control group to one. The results are presented as the mean and standard deviation from three independent transfections. *, P value<0.05. C) The conditioned medium was collected from each treatment group and processed for gelatin zymography to determine the expression of MMP-9 in the conditioned medium. A representative image is shown from two independent transfections.

Since PP2 paradoxically rescued SFK activation and induction of MMP-9 and ICAM-1 by TNF-α in Thy-1+ MEFs, we utilized another SFK selective inhibitor, SU6656 to determine the role of SFK activation in TNF-α-activated gene expression in MEF Thy-1 subsets [30]. SU6656 (20 µM) substantially diminished the TNF-α-activated expression of MMP-9 and ICAM-1 in both Thy-1 subsets (Figure 5, A and B). Furthermore, elevated MMP-9 expression in the conditioned medium in response to TNF-α was nearly abrogated by SU6656 in Thy-1− MEFs (Figure 5C). In contrast to PP2, SU6656 not only failed to activate SFK in Thy-1+ MEFs, but also abrogated TNF-α-mediated SFK activation in Thy-1− MEFs (Figure 5D). In accord, tyrosine phosphorylation of SFK-associated FAK, was stimulated only in Thy-1− MEF and abolished by SU6656 (Figure 5D). To confirm the requirement of SFK for TNF-α-activated gene expression in MEFs, dominant-negative Fyn (dnFyn), a member of SFK family, was expressed by transient transfection to specifically interfere with SFK activation in Thy-1− MEFs exposed to TNF-α. dnFyn expression substantially reduced induction of MMP-9 and ICAM-1 mRNA by TNF-α, as a more than 50% decrease was observed when compared to the Thy-1− MEFs transfected with a backbone vector (Figure 6A). Similar to Thy-1 expression (Figure 2), dnFyn expression attenuated TNF-α-mediated activation of the IL-8 promoter in Thy-1− MEFs (Figure 6B). Moreover, dnFyn expression reduced induction of MMP-9 in the conditioned medium collected from TNF-α-stimulated Thy-1− MEFs (Figure 6C). These results demonstrated a requirement for SFK activation in TNF-α-activated gene expression in MEF Thy-1 subset.

**Figure 7:**
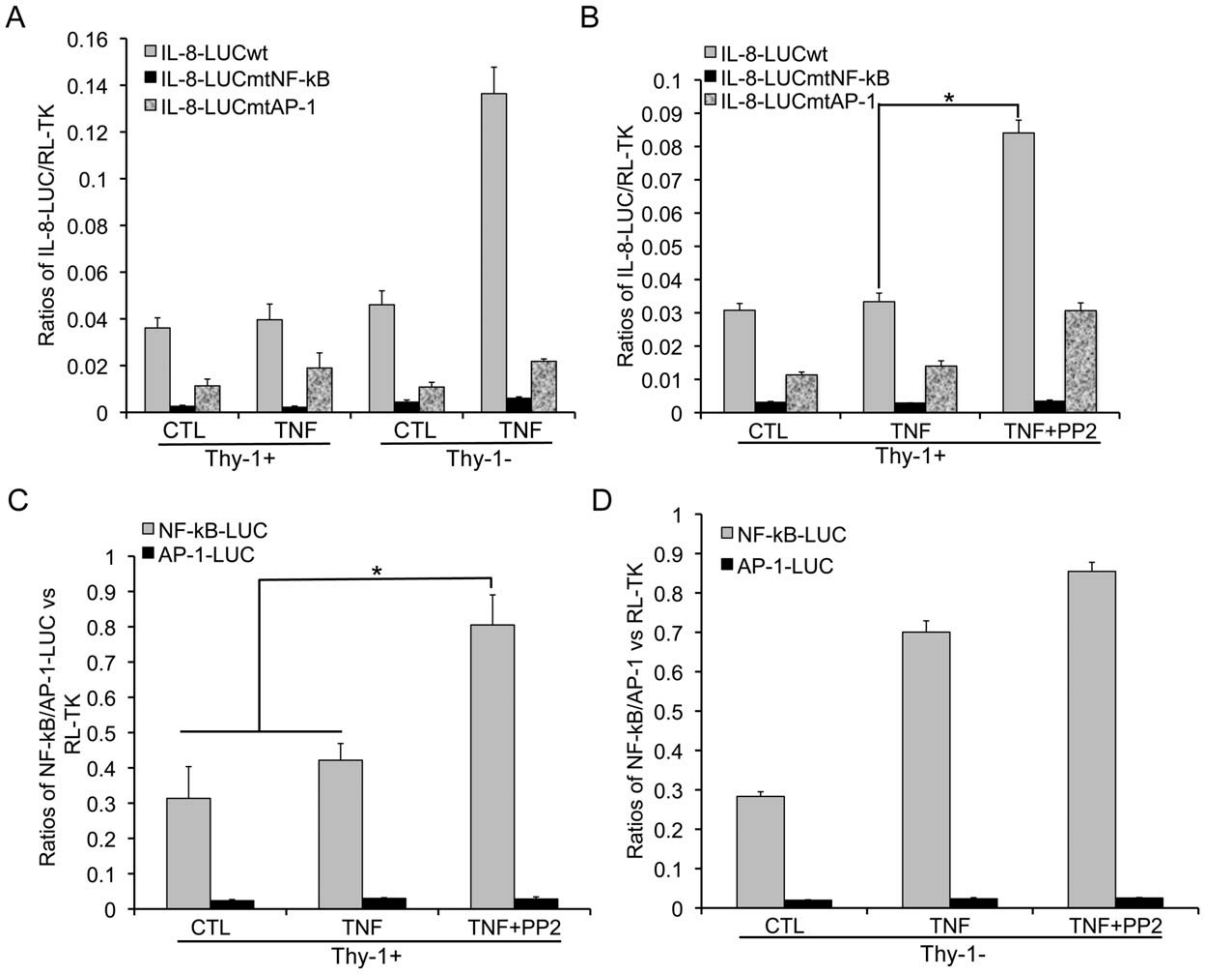
Activation of NF-κB-mediated gene expression by PP2 in Thy-1+ MEFs. A) MEF Thy-1 subsets were transfected with IL-8-LUC reporter (IL-8-LUCwt) or its variants with either the NF-κB or the AP-1 binding sites mutated (IL-8-LUCmtNF-κB or IL-8-LUCmtAP-1). The reporter activity was examined upon exposure to TNF-α. The ratios of IL-8-LUC variants vs. the cotransfected RL-TK were compared across the groups. B) Similar to part A except that the activity of IL-8-LUC variants was examined in Thy-1+ MEFs exposed to TNF-α ± PP2. C) Similar to part B except that activity of NF-κB-LUC and AP-1-LUC was examined in Thy-1+ MEFs exposed to TNF-α ± PP2. D) Similar to part C except that the experiments were carried out in Thy-1− MEFs. The results are presented in mean and standard deviations obtained from at least three independent transfections preformed in duplicates. * indicates a P value<0.05.

Requirement of the NF-κB binding site for PP-2-mediated gene activation in response to TNF-α – To investigate the role for NF-κB and AP-1 in differential gene activation by TNF-α in Thy-1 subsets of MEFs, we examine the IL-8 promoter activity with or without mutations in the NF-κB or AP-1 binding site using reporter assays. In both Thy-1 subsets, mutational inactivation of the NF-κB binding site nearly silenced the IL-8 promoter as the IL-8-LUCmtNF-κB exhibited less than 10% activity of the IL-8-LUCwt (Figure 7A). Moreover, loss of the NF-κB binding site abrogated activation of the IL-8 promoter by TNF-α in Thy-1− MEFs. On the other hand, IL-8-LUCmtAP-1 retained around 30% activity of the IL-8-LUCwt and exhibited around 2-fold increase upon exposure to TNF-α in both MEF subsets. These findings implicate that NF-κB binding mediated transcriptional activation plays a more critical role in differential activation of the IL-8 promoter by TNF-α in Thy-1 subsets of MEFs. Because PP2 rescued TNF-α-activated gene expression in Thy-1+ MEFs (Figure 4), we set to determine the role for NF-κB and/or AP-1 binding sites in PP2-mediated rescue of the IL-8 promoter activation. Consistent with activation of the TNF-α responsive endogenous genes MMP-9 and ICAM-1, PP2 rescued activation of the IL-8 promoter by TNF-α because only the simultaneous exposure to TNF-α and PP2 resulted in a around 3-fold increase in the IL-8-LUCwt reporter activity over the control group (Figure 7B). PP2 failed to activate the IL-8-LUCmtNF-κB in which NF-κB binding was inactivated. In contrast, the IL-8-LUCmtAP-1 reporter was activated by a combination of PP2 and TNF-α although the elevated activity of IL-8-LUCmtAP-1 remained lower than that of IL-8-LUCwt. These findings suggest that the NF-κB binding site is essential to PP2-mediated rescue of activation of the IL-8 promoter in Thy-1+ MEFs. To confirm the link between NF-κB binding to the promoter and PP2 in Thy-1+ MEFs, we examine the effects of PP2 on the luciferase reporters that are regulated by tandem multiple copies of NF-κB or AP-1 binding sites, respectively. Consistent with the response of the IL-8 promoter variants to PP2, NF-κB-LUC, but not AP-1-LUC was activated by PP2 in Thy-1+ MEFs exposed to TNF-α (Figure 7C). Similar to the disparate contribution of NF-κB and AP-1 binding to activation of the IL-8 promoter by TNF-α, only NF-κB-LUC, but not AP-1-LUC, was significantly activated by TNF-α in Thy-1−MEFs (Figure 7D). Taken together, these findings indicate a critical role for NF-κB binding in differential gene activation by TNF-α in Thy-1 subsets of MEFs.

## Discussion

Thy-1 fibroblast subsets appear to behave differently in response to the inflammatory mediator TNF-α. The current study investigates the role of Thy-1 in TNF-α-activated gene expression in MEFs. Our findings indicate that Thy-1+ MEFs exhibit reduced gene activation, namely MMP-9, ICAM-1 in response to TNF-α, and a reporter gene controlled by the human IL-8 promoter, when compared to Thy-1− MEFs. Moreover, TNF-α activates SFK in Thy-1− MEFs, but not in Thy-1+ MEF. Pharmacologic activation of SFK rescues TNF-α-activated gene expression in Thy-1+ MEFs, whereas blockade of SFK activation abrogates TNF-α-activated gene expression in Thy-1− MEFs. Taken together, these findings suggest that Thy-1 attenuates TNF-α-activated gene expression via interfering with SFK activation in fibroblasts, which may contribute to the distinct roles of Thy-1 fibroblast subsets in response to inflammation.

A large body of evidence indicates that Thy-1 expression in fibroblasts modulates inflammation [1], [2], [3]. One such paradigm has been illustrated by the predominance of Thy-1− lung fibroblasts in pulmonary fibrosis accompanied by inflammation. Moreover, Thy-1−/− mice exhibit a marked increase in the severity of lung fibrosis consequent to injury from intratracheal administration of bleomycin [19]. Our findings reveal that MEF Thy-1 subsets differ in TNF-α-activated gene expression (Figure 1). By introducing Thy-1 into Thy-1− MEFs, we demonstrate that Thy-1 expression is sufficient to attenuate TNF-α-activated gene expression (Figure 2). Similarly, TNF-α-activated MMP-9 expression is robust in the Thy-1− RFL6.EV while absent in RFL6.Thy-1, although RFL6.Thy-1 cells exhibit greater basal expression of MMP-2 and MMP-9 than the RFL6.EV (Figure 2, D & E). Nevertheless, the results from RFL6 variants are fundamentally in line with that from MEFs, which strongly suggests that attenuation of TNF-α-activated gene expression by Thy-1 is a reproducible phenomenon in fibroblasts isolated from different species. Given the roles of MMP-9 in cell migration and ICAM-1 in cell-cell interaction, it is conceivable that Thy-1 modulates mobilization of fibroblasts by TNF-α in inflammation. In support of this notion, TNF-α induces invasion only in the Thy-1− MEFs despite greater basal level of invasive potential in Thy-1+ MEFs (Figure 1D). Higher baseline of invasion in Thy-1+ MEFs might be attributed higher basal levels of MMP-2 at the mRNA and protein levels (Figure 1 and data not shown). Reduced activation of the human IL-8 promoter in the presence of Thy-1 expression lends further support to this concept and suggests that Thy-1 expression inhibits TNF-α-mediated transcriptional activation (Figure 2). Our findings are in line with a previous study that indicates Thy-1− fibroblasts are more sensitive to IL-1β than their Thy-1+ counterparts, which was manifested by greater induction of IL-6 in Thy-1− fibroblasts [17]. Importantly, TNF-α and IL-1β are key cytokines that mediate injury inflammation and the subsequent fibrosis in experimental lung fibrosis triggered by bleomycin. Moreover, Thy-1 limits the ability of fibroblasts to activate latent TGF-β1, a pivotal modulator of inflammation [18]. Our findings reinforce the idea that Thy-1 expression in fibroblasts modulates their responses to inflammatory mediators, which may account for the more severe lung fibrosis in Thy-1 deficient mice and the predominance of Thy-1− fibroblasts in the fibroblastic foci in patients with idiopathic pulmonary fibrosis [19].

Activation of NF-κB is central to TNF-α-mediated cellular responses. A comparison of the transcriptional activity of the IL-8 promoter and its variants with inactivation of either NF-κB or AP-1 binding site implicates a greater role for NF-κB in TNF-α-activated gene expression than AP-1 (Figure 7). Furthermore, NF-κB appears to be responsible for differential TNF-α-activated gene expression between Thy-1 subsets because NF-κB-LUC but not AP-1-LUC is activated by TNF-α in Thy-1− MEF. In accord, PP2 rescues activation of IL-8-LUCwt and IL-8-LUCmtAP-1 which harbor the intact NF-κB binding site, but not IL-8-LUCmtNF-κB in Thy-1+ MEFs (Figure 7). Notably, TNF-α-induced degradation of I-κBα, a pivotal step for activation of NF-κB, is comparable between MEF Thy-1 subsets (Figure 3). This piece of evidence indicates that Thy-1 modulates NF-κB activity in an I-κBα independent manner. Our findings are reminiscent of a previous observation that reveals equivalent receptor expression and surface binding to IL-1β as well as the consequent NF-κB activation in the rat lung fibroblast Thy-1 subsets, albeit greater IL-6 induction by IL-1β is observed in Thy-1− subset [17]. In summary, these results suggest that the transcriptional activity of NF-κB is regulated by Thy-1 in fibroblasts after release from its inhibitor I-κBα. The transcriptional activity of NF-κB might be negatively regulated by Thy-1 via inhibition of SFK because PP2 rescues NF-κB-dependent gene activation and SFK activation by TNF-α in Thy-1+ MEFs (Figures 3–7).

Our attempts to determine the Thy-1-modulated TNF-α signaling pathway(s) have led to an unexpected finding in that a commonly used SFK selective inhibitor PP2 displays minimal effect on SFK activation as well as TNF-α-activated gene expression in Thy-1− MEFs (Figures 3 & 4). In stark contrast to its reported inhibition of SFK activation, not only did PP2 fail to inhibit SFK and gene activation by TNF-α in Thy-1− MEFs, it paradoxically rescued SFK activation and gene expression by TNF-α in Thy-1+ MEFs (Figures 3 & 4). Consistent with the remainder of our findings, another SFK selective inhibitor SU6656 substantially reduced SFK activation and gene expression by TNF-α in Thy-1− MEFs (Figure 5). The opposing effects of the two SFK selective inhibitors, SU6656 and PP2, on the Thy-1 subsets concur in regard to the conclusion that SFK activation is critical for TNF-α-activated gene expression in MEFs. Indeed, the requirement of SFK activation for TNF-α-activated gene expression in MEFs is further supported by a molecular SFK-specific inhibitory approach. Expression of dominant-negative Fyn significantly reduced gene activation by TNF-α in Thy-1− MEFs (Figure 6). Thy-1 appears to down-regulate TNF-α-activated gene expression via interfering with SFK activation. The unexpected effect of PP2 on SFK activity could indicate that Thy-1 employs an unidentified PP2-sensitive signaling pathway to inhibit SFK activation and consequently the cellular responses to TNF-α in fibroblasts. This speculation is supported by a recent report that demonstrates PP2-mediated increase in phosphorylation at the positive regulatory site Tyr-416 in Src and decrease in phosphorylation at the negative regulatory site Tyr-529 [31]. The report further suggests that the kinase Csk is the target of PP2 in activation of Src because Csk phosphorylates Src at Tyr-529 and its catalytic activity is inhibited by PP2. In line with this postulate, Thy-1 exists in a multiple protein complex that contains both Csk and Fyn, a SFK family member [2]. Expression of dominant-negative Fyn abrogates TNF-α-activated gene expression (Figure 6). Based on our results and others' findings, we propose that Thy-1 inhibits SFK, very likely Fyn, by promoting Csk activity and PP2 rescues SFK activation by inhibiting Csk in Thy-1+ MEFs. These findings warrant further investigation of the functional interaction among Thy-1, Csk, and SFK in fibroblast in the context of TNF-α-induced inflammatory response. The unknown PP2-sensitive signaling pathway might be dependent on specific cell or species type because PP2 abolishes SFK and the consequent NF-κB activation by TNF-α in human epithelial cell lines [23], [24]. Exogenous expression of Thy-1 on Thy-1-nonexpressing fibroblasts results in inhibition of SFK activity and subsequent activation of RhoA, leading to focal adhesion assembly and stress fiber formation [5], [27], [28], [29]. The cellular context dependent modulation of SFK by Thy-1 is further illustrated by a previous report that suggests Thy-1 is pivotal for thrombospondin-1-mediated SFK activation as well as focal adhesion disassembly in a SFK-dependent manner [5]. The apparently conflicting modulation of SFK by Thy-1 highlights the complexity of Thy-1 mediated signaling regulation and warrants further investigation of the molecular determinants of Thy-1 actions, such as its linkage with glycophosphatidylinositol and association with lipid rafts [1], [2], [3]. The unexpected findings of PP2-mediated activation of SFK in Thy-1+ MEFs raise caution in the design and interpretation of experiments involving small molecule inhibitors of SFK.

In summary, the current study reveals greater SFK activation and gene expression in response to TNF-α in Thy-1− MEFs compared with their Thy-1+ counterparts. Our results further suggest that Thy-1 down-regulates TNF-α-activated gene expression by interfering with SFK activation and NF-κB-mediated transactivation. These findings provide a novel mechanistic insight to the distinct roles of Thy-1 subsets of fibroblasts in inflammation and warrant further investigation of Thy-1-mediated regulation of the TNF-α-SFK-NF-κB network in fibroblasts residing within various tissues upon inflammation.

## Materials and Methods

### Reagents and Antibodies

Protease Inhibitor Cocktail and Phosphatase Inhibitor Cocktail 2 were purchased from Sigma (St. Louis, MO). The SFK selective inhibitors PP2 and SU6656 were purchased from Calbiochem (San Diego, CA). Recombinant mouse TNF-α and human TGF-β1 were obtained from Peprotech (Rocky Hill, NJ). The reporter constructs that express firefly luciferase regulated by the human IL-8 promoter either inact (IL-8-LUCwt) or with the NF-κB or AP-1 binding elements mutated (IL-8-LUCmtNF-κB and IL-8-LUCmtAP-1) were kindly provided by Dr. Gary Wu at the University of Pennsylvania School of Medicine [26]. The luciferase reporters that are regulated by multiple copies of either NF-κB or AP-1 binding sites (NF-κB-LUC and AP-1-LUC) were purchased from Promega. The pRL-TK vector expressing Renilla luciferase driven by the thymidine kinase promoter was obtained from Promega (Madison, WI). The dominant-negative Fyn mutant (the K229M mutant) expressing vector (dnFyn) and the backbone vector pCMV5 were kindly provided by Dr. Marylin Resh at the Memorial Sloan Kettering Cancer Center [32]. Thy-1 expression vector and the backbone vector pCDNA3.1.Zeo were generated as previously described [5]. Rabbit polyclonal antibodies specific for SFK, phospho-tyrosine, and a β-actin-specific goat polyclonal antibody were obtained from Santa Cruz Biotechnology (Santa Cruz, CA). Rabbit polyclonal antibodies specific for c-Src with tyrosine 416 phosphorylated (or other SFK members with equivalent sites phosphorylated) and specific for I-κBα were purchased from Cell Signaling Technology (Beverly, MA). A rabbit polyclonal antibody specific for Focal Adhesion Kinase (FAK) was purchased from Upstate (Lake Placid, NY). FITC-labeled antibodies against Thy-1.1 and Thy-1.2 were purchased from BD Biosciences (Bedford, MA).

### Primary Fibroblasts Purification, Cell Sorting and Culture

Mouse embryonic fibroblasts (MEF) from C57 mice were isolated and sorted with FACS using FITC-labeled Thy-1-specific antibodies as previously described [15]. The purity of each subset was above 95%. The sorted MEFs were cultured in DMEM (Sigma, St. Louis, MO) supplemented with 10% FBS (vol/vol), 100 µg/ml penicillin and 100 µg/ml streptomycin in a humidified incubator with 5% CO2. Two different batches of sorted Thy-1 subpopulations of MEFs were employed in this study and yielded similar results. Two variant rat fetal lung fibroblast transfectants (RFL6) were generated by introducing either the backbone vector or the murine Thy-1.2 cDNA expression vector into RFL6 Thy-1− cells as previously described [5] and hence were named as RFL6.EV and RFL6.Thy-1, respectively. The RFL6 transfectants were cultured in F12K (Invitrogen, Carlsbad CA) supplemented with 10% FBS (vol/vol), 100 µg/ml penicillin, 100 µg/ml streptomycin and the selecting reagent Zeocin (Invitrogen, Carlsbad, CA). All the fibroblasts were serum-starved in the appropriate medium containing 0.5% FBS for 48 hours prior to exposure to the indicated doses of TNF-α or TGF-β1. In selected experiments, the MEFs were pre-treated with PP2 (10 µM) or SU6656 (20 µM) for 30 minutes followed by exposure to TNF-α (5 ng/ml) for the indicated duration.

### RNA Isolation, Reverse Transcription and Real-time PCR

Quantitative RT-PCR was performed to determine mRNA levels of Genes Of Interest (GOI) in MEFs subject to the indicated treatments as previously described [33]. The primers used were as follows: mouse 36B4 (GenBank BC011106, conserved in rat 36B4), forward primer (nucleotides 69–88), 5′-CGACCTGGAAGTCCAACTAC-3′, and reverse primer (nucleotides 177–160) 5′-ATCTGCTGCATCTGCTTG-3′; mouse ICAM-1 (GenBank NM_010493), forward primer (nucleotides 981–1000), 5′-GACGCAGAGGACCTTAACAG-3′, and reverse primer (nucleotides 1119–1102), 5′-GACGCCGCTCAGAAGAAC-3′; mouse MMP-9 (GenBank BC046991), forward primer (nucleotides 2033–2052), 5′-CCTGGAACTCACACGACATC-3′, and reverse primer (nucleotides 2261–2244), 5′-GGTGGTGGTGGTGGTCTC-3′. The ratio of GOI/36B4 was compared among samples and the fold change of GOI expression was obtained by setting the values from the untreated cells to one.

### Gelatin Zymography

Serum-starved fibroblasts were exposed to the indicated doses of TNF-α or TGF-β1 with or without pretreatment with PP2 (10 µM) or SU6656 (20 µM) for 30 minutes. The conditioned medium was collected at 48 hours after the specified treatments and processed for gelatin zymography as described elsewhere [33]. The intensity of MMP-9 gelatinlytic zones on the gels was quantified by densitometry using the AlphaImager 2200 Documentation Analysis System with AlphaEase StandAlone Software Version 5.04 (Alpha Innotech Corp., San Leandro, CA).

### Invasion Assays

The invasive potential of TNF-α-stimulated MEFs was assessed using the BD Biocoat Invasion System (BD Biosciences, Bedford MA) following provided instructions. Briefly, serum-starved MEF cells were seeded into the top chamber at a density of 1×10^4^ cells per well. DMEM containing 5% FBS was added into the bottom chamber as a chemoattractant. TNF-α (5 ng/ml) was added at 16 hours after seeding and left in the medium for another 24 hours. The non-invading cells on the top surface of the membrane were removed by scrubbing twice with a swab. The invading cells on the bottom surface of the membrane were stained using Hema 3 system (Fisher Scientific, Middletown, VA). Invading cells were enumerated under light microscopy. The invasion assays were performed in triplicates for each treatment group. Results shown were the average from three triplicates.

### Immunoblots

The immunoblots for signaling molecules were carried out as previously described [34]. Fibroblasts subject to the indicated treatments (TNF-α 5 ng/ml ± PP2 or SU6656) were lysed in 1× Laemmli buffer. An equal amount of total protein from each lysate was fractionated in an 8–16% gradient Tris-glycine Precast Gel (Invitrogen) and transferred to a PVDF membrane (Bio-rad). The blots were probed for protein levels of SFK, phospho-SFK and I-κBα using the appropriate antibodies (anti-SFK, 1∶500; anti-phospho-SFK, 1∶1000; anti-I-κB-α, 1∶1000). To monitor the loading variations, the blots were stripped and re-probed for β-actin. The abundance of tyrosine-phosphorylated FAK associated with SFK was measured by immunoblots using phosphor-tyrosine-specific antibody (anti-Tyr-P, 1∶500) on immunoprecipitates by SFK-specific antibody as described in *in vitro* SFK kinase assay. The total SFK-associated FAK was evaluated by immunoblot with a FAK-specific antibody after stripping the blot.

### *In vitro* SFK kinase assay

SFK catalytic activity in treated MEFs was analyzed using the Src Assay Kit (Upstate, Lake Placid, NY) as per the provider's instructions. The cell extracts were prepared from the MEFs subject to the indicated experimental conditions and immunoprecipitated with an SFK-specific antibody as described previously [35]. Briefly, MEFs were pretreated with PP2 (10 µM) for 30 minutes followed by exposure to TNF-α (5 ng/ml) for another 5 minutes. The treated MEFs were then lysed in EBCD buffer (50 mM Tris, pH 8.0, 120 mM NaCl, 0.5% Nonidet P-40, 5 mM DTT). One milligram of each lysate was pre-cleared with BSA/Protein G-agarose slurry (Invitrogen) followed by immunoprecipitation with 1 µg of SFK-specific antibody. The phosphotransferase activity of SFK in immunprecipitates was evaluated by measuring the [γ-32P] ATP incorporated into an SFK-specific peptide substrate. The SFK activity for each sample was determined after corrected with the values of immunoprecipitates by a control rabbit IgG per recommendations in the provided manual.

### Transient Transfection and Luciferase Reporter assays

Transient transfection and luciferase reporter assays were carried out as previously described [33]. Briefly, MEFs were seeded into a 24-well culture plate at a density of 5×10^4^ cells per well. On the next day, the two luciferase reporters IL-8-Luc and pRL-TK were co-transfected into MEFs along with either the backbone vectors or the vectors express mouse Thy-1 or dnFyn using Lipofectamine 2000 (Invitrogen, Carlsbad, CA). The cells were serum-starved for 24 hours after transfection and then exposed to TNF-α (5 ng/ml) for 6 hours. The treated cells were harvested and assayed for luciferase activity using the Lumat LB 9507 Luminometer (Berthold, Australia) along with the Dual-Luciferase Reporter Assay System (Promega) per the provider's instructions. Variation in transfection efficiency was monitored and corrected with the co-transfected pRL-TK. The ratio of Fire Fly luciferase activity to Renilla luciferase activity in each sample served as a measurement of normalized luciferase activity. The fold change of normalized luciferase activity was obtained by setting the values from the untreated cells at one. To determine the effects of Thy-1 and dominant-negative Fyn on TNF-α-activated gene expression, Thy-1− MEFs were transfected with either the backbone vectors or Thy-1 or dn-Fyn vectors followed by exposure to TNF-α. Total cell RNA (24 hr post-exposure to TNF-α) or the conditioned medium (48 hrs post-exposure to TNF-α) was collected to determine the expression of MMP-9 and ICAM-1. To determine effects of PP2 on activity of the human IL-8 promoter, IL-8-LUC and its variants with mutational inactivation of the NF-κB and AP-1 binding elements were transfected into MEF Thy-1 subsets in the presence of TNF-α (5 ng/ml) ± PP2 (10 µM). The transcriptional activity of NF-κB and AP-1 was assessed by transfecting Thy-1+ MEFs with NF-κB-LUC and AP-1-LUC in the presence or absence of TNF-α (5 ng/ml) ± PP2 (10 µM).

### Statistical Analyses

The results that were obtained from at least three independent experiments were presented as mean±standard deviation. Statistical evaluation was performed between individual treatment groups by unpaired two-tailed Student's T-test using GraphPad Prism (GraphPad Software, version 3). A P value of less than 0.05 was considered significant.
